# Effect of five non-invasive treatments on body composition, physical function and quality of life in elderly sarcopenia: a network meta-analysis of 22 randomized controlled trials

**DOI:** 10.3389/fphys.2025.1610138

**Published:** 2025-08-25

**Authors:** Demin Kong, Jiangang Chen

**Affiliations:** ^1^ Graduate School, Kyungnam University, Changwon, South Gyeongsang, Republic of Korea; ^2^ College of P.E and Sports, Beijing Normal University, Beijing, China

**Keywords:** sarcopenia, rehabilitation, physical therapists, exercise, resistance training, electric stimulation, proteins

## Abstract

**Introduction:**

While exercise interventions are widely used for sarcopenia management, the comparative efficacy of different non-invasive treatments remains unclear. This network meta-analysis evaluated five interventions (aerobic training, resistance training, aerobi-resistance training, whole-body electrical stimulation, and electrical stimulation with protein supplementation) on body composition, physical function and quality of life in elderly sarcopenia patients.

**Methods:**

Six databases, including PubMed, Embase and Web of Science, were systematically searched, and 22 randomized controlled trials with a total of 1062 elderly patients with sarcopenia were finally included. The outcome indicators were those related to the evaluation of body composition, physical function and quality of life. Net meta-analysis was performed using Stata 17.0 to assess the relative effectiveness of each intervention and to test the consistency of direct and indirect evidence.

**Results:**

ES&P (SMD = −3.33, 95% CI [−4.23, −2.44], p < 0.00001) and AT (SMD = −1.31, 95% CI [−1.83, −0.79], p < 0.00001, I^2^ = 58%) demonstrated significant effects in terms of fat reduction, RT achieved a significant effect in terms of muscle gain (SMD = 0.50, 95% CI [0.08, 0.91], p < 0.05, I^2^ = 42%), RAT was the most effective in terms of strength gains (SMD = 0.51, 95% CI [0.05, 0.98], p < 0.05, I^2^ = 0%), and RAT also demonstrated a favorable effect in terms of improving quality of life (SMD = 1.42, 95% CI [0.13, 2.70], p < 0.05, I^2^ = 55%).

**Conclusion:**

ES & P and AT have good effect on fat reduction, RT has the best effect on increasing muscle, RAT is the most effective in improving strength, and AT is the best in improving quality of life. Different treatments have different effects on functional indicators, and clinics should personalize the selection of different interventions according to the patient’s condition and combine multiple interventions to achieve the best recovery results.

## 1 Introduction

Sarcopenia is a progressive musculoskeletal disorder characterized by the loss of muscle mass and strength, particularly prevalent in aging populations (Cruz-Jentoft et al., 2019; Cruz-Jentoft and Sayer, 2019; Papadopoulou, 2020). The condition significantly impairs physical function and increases vulnerability to adverse health outcomes, making early intervention critical (Gielen et al., 2023; Lu et al., 2021; Bauer et al., 2019; Sayer et al., 2024).

The main pathologic feature of patients with sarcopenia is the reduction of skeletal muscle mass and function, of which the loss of muscle mass is the most significant and clinically meaningful indicator. Several studies have shown that decreased muscle mass is the main pathophysiologic feature of sarcopenia and is closely associated with poor prognosis and increased mortality. For example, Walston (2012) found in a large-scale epidemiologic study that decreased muscle mass was directly associated with mortality, decreased quality of life, and loss of self-care in the elderly. Decreased muscle mass in patients with sarcopenia also has a serious impact on their activities of daily living, affecting their gait, balance, and physical function. Studies have shown that patients with sarcopenia have an unstable gait and slow gait speed, often accompanied by dependence on daily activities (Coletta and Phillips, 2023). A study by Perez-Sousa et al. (2019) noted that sarcopenia is associated with an unsteady gait, slowed gait speed, and falls, which can have a serious impact on the patient’s functional independence. In addition to reduced muscle mass and strength, impaired physical function is an important aspect of the clinical presentation of patients with sarcopenia. Studies have shown that patients with sarcopenia have reduced ability to perform daily activities, especially lower limb muscle strength, which significantly affects their basic functions such as walking, standing, and stair climbing. Abellan van Kan et al. (2012) found that lower limb muscle strength loss in sarcopenia patients was closely associated with impaired balance, increased risk of falls, and gait abnormalities. In terms of quality of life, the impact of sarcopenia on patients is particularly serious. Studies have shown that the loss of muscle strength not only affects the athletic ability of patients, but also has a direct impact on their social activities and psychological status, resulting in psychological problems such as low mood and depression. For the elderly, the lack of physical strength and limitation of daily activities caused by sarcopenia is usually accompanied by a significant decline in quality-of-life Chow et al. (2020) found that sarcopenia is closely associated with a decline in quality of life, poorer health and psychological distress.

Currently, common methods of treating sarcopenia include pharmacological treatment, nutritional intervention, physiotherapy and exercise training. Pharmacological treatment is mainly aimed at improving muscle quality and function, such as the use of anti-aging drugs, insulin-like growth factors, hormonal drugs. Emerging pharmacological approaches, such as myostatin inhibitors and selective androgen receptor modulators, are also under investigation to enhance muscle mass and strength in sarcopenia patients (Lee, 2023) (Fonseca et al., 2020). Some studies have already shown that pharmacological interventions can improve muscle quality to a certain extent and slow down the progression of sarcopenia (Caballero-García et al., 2021). In terms of nutritional intervention, supplementation with nutrients such as protein and vitamin D has been shown to have a positive effect on improving muscle function (Papadopoulou et al., 2021). Exercise training, especially resistance training, has been widely recognized as the most effective non-pharmacological treatment. Studies have shown that resistance training can effectively increase muscle strength and improve physical function in elderly patients with sarcopenia. After regular resistance training, muscle strength increased significantly and gait balance function was improved in elderly patients (Giallauria et al., 2016). Recent studies demonstrate that neuromuscular electrical stimulation (NMES) can effectively improve muscle strength and physical performance in sarcopenic patients (Pring et al., 2021). However, standardized protocols and long-term efficacy still require further investigation.

This network meta-analysis of 22 randomized controlled trials systematically compares the efficacy of five key non-invasive interventions in improving body composition, physical function, and quality of life in elderly sarcopenia patients. Given the high prevalence of sarcopenia in the elderly population and its profound impact on quality of life, this study provides urgently needed evidence to guide clinical decision-making, optimize treatment strategies, and inform rehabilitation guidelines for this growing population.

## 2 Methods

This study was guided by the Preferred Reporting Items for Systematic Evaluation and Meta-Analyses (the PRISMA list for NMAs10 and the Cochrane Handbook for the Evaluation of Intervention Systems) (Hutton et al., 2015). Registration number: CRD42024612972.

### 2.1 Data sources

Systematic searches were conducted in PubMed, Embase, Web of Science, Cochrane, EBSCO, and China National Knowledge Infrastructure (CNKI), and the selection of included studies was done independently by 2 researchers (XL, HL). Searches were performed in PubMed and Cochrane using terms from MeSH. Searches were performed in Embase using terms in Emtree and in CNKI using subject terms combined with free terms. The reference lists of relevant articles were also manually screened for other studies that might be eligible. The search timeframe was from January 2010 up to October 2024, and was limited to human studies published in Chinese or English, and only core journals were included in Chinese.

The search strategy followed the PICOS principles of evidence-based medicine (Amir-Behghadami and Janati, 2020): (P) population: elderly patients with sarcopenia; (I) interventions: aerobic training, resistance training, resistance aerobic training, whole-body electrical stimulation (WB-EMS) and whole-body electrical stimulation plus protein supplementation (ES&P); (C) Control group: The control group had no therapy or other non-invasive treatments; (O) Results Body composition (Skeletal Muscle) was assessed by ASM (appendicular skeletal muscle mass), TSM (total skeletal muscle mass), FFM (fat-free mass) and SMI (Skeletal Muscle mass Index); PBF (Percentage body fat), TFM (total fat mass), BFM (body fat mass) and FM (fat mass) were selected for body composition; Strength, grip strength and handgrip strength were selected for body function. Quality of life was evaluated using objective physical function measures that directly impact daily living activities: gait velocity, maximum walking speed, and 2-min walk distance. These parameters serve as well-established physical proxies for QoL in elderly sarcopenia patients, as they correlate strongly with independence in basic and instrumental activities of daily living; (S) Study type: RCTs.

### 2.2 Study selection

As an example, the PubMed database was searched using several medical search terms, including “exercise” [Mesh], “training” [Mesh], and “sarcopenic” to ensure that literature related to exercise, training and sarcopenia was retrieved. These search terms allowed for extensive coverage of research related to the field. To further ensure the comprehensiveness and accuracy of the literature, the reference lists of relevant articles were also manually screened for relevant studies that may have been missed.

After obtaining the initial literature, a rigorous screening process was carried out on this literature. Firstly, an automatic duplicate literature check was performed using EndNote software to eliminate duplicate records in the database that may have arisen due to different search strategies or data sources. Subsequently, duplicates that were not automatically identified during the screening process were further removed by manually reading the titles and abstracts to ensure the uniqueness and representativeness of the screened literature. For the remaining literature, a more rigorous review was conducted. The following types of studies were mainly excluded: studies in non-elderly sarcopenia patient groups, studies that did not assess relevant indicators, and studies that did not use non-invasive methods of intervention. In addition, review articles, conference abstracts, animal studies, research protocols, case reports, retrospective studies, and book chapters were also excluded because they often lacked sufficient primary data or scientific validity to provide specific analyses and conclusions about the effects of interventions. These stringent literature screening criteria ensured that the final included studies could provide high-quality evidence to support this study and further enhance the scientific validity and credibility of the study.

### 2.3 Eligibility criteria

Randomized clinical trials (RCTs) in people with confirmed sarcopenia comparing the effects of different non-invasive treatments were included.

Studies were eligible for inclusion if they met the following criteria: 1) they were RCTs; 2) they were obese elderly patients with sarcopenia; 3) they were non-invasive treatments; 4) data on outcome metrics were complete; and 5) the experimental group interventions were aerobic training, resistance training, resistance aerobic training,WB-EMS and ES&P, and no intervention or other non-invasive method in the control group; 6)Measure at least one of the following: ASM, TSM, FFM, SMI, PBF, TFM, BFM, FM, strength, grip strength, handgrip strength, GV, MWS usual walking speed and 2 min-walk.

Studies were excluded if they: 1) were non-RCTs 2) were experimental animal studies, review-type literature, conference reports, case reports, letters, and repetitive publications, etc.; 3) were not available in full text; 4) had incomplete data on experimental results or data metrics that could not be extracted; 5) did not report relevant metrics of interest to the study; 6) had patients with motor system disorders other than sarcopenia; 7) Non-core journal literature in Chinese published literature.

### 2.4 Data collection

The literature obtained was screened by 2 researchers by importing the collected literature into EndNote 20 software according to the search strategy. Duplicate literature was first excluded and then titles and abstracts were read for initial screening. The remaining literature was further screened by reading the full text in detail according to the inclusion and exclusion criteria. Subsequently, 2 researchers cross-checked the results of their respective screening, and if the checking was consistent, the study was included; if there was any disagreement, the third researcher was consulted, and the final inclusion of the study was made after the discussion reached a consensus.

For eligible trials, 2 trained researchers independently extracted data from the included literature using a standardized data extraction form and generalized the risk of risk bias. The extracted data mainly included 1) basic information about the included literature (first author, year of publication, country, etc.); 2) demographic characteristics of the subjects (number of people in the experimental and control groups, age, and gender); 3) details of the interventions (type of intervention, intensity, duration, and frequency); and 4) outcome metrics (mean and standard deviation, with the primary outcome metrics chosen to include ratings for both body composition (skeletal muscle) and measures of body composition (adiposity); secondary outcome measures were selected to include measures of body function (strength) and quality of life. For studies in which results were presented graphically without numerical summaries, numerical data were extracted for analysis using a validated plot digitization tool (Get Data 2.22). Corresponding authors were contacted for additional information when required.

### 2.5 Risk of bias of the systematic review

All eligible studies were assessed for risk of bias (ROB) by two researchers independently, according to the Cochrane 5.1 version of the Risk of Bias Assessment Tool (Sterne et al., 2019). Any discrepancies in their assessments were resolved through discussion, and if consensus could not be reached, a third researcher was consulted to make the final decision. The tool includes seven domains: random sequence generation, allocation concealment, blinding of participants and personnel, blinding of outcome assessors, incomplete outcome data, selective reporting, and other biases. Risk assessment analyses were performed using Review Manager 5.3 (Nordic Cochrane, Denmark), and each domain was rated as “unclear,” “low risk,” or “high risk, we categorized the overall risk of bias for each study as 1) low ROB: there were no domains assessed as high risk, and there may have been domains assessed as unclear but fewer than three; 2) medium ROB: there was a domain assessed as high risk but no more than one; or there were no domains of high risk but more than three domains assessed as unclear; and 3) high ROB: all domains other than the above are classified as high risk.

### 2.6 Statistical analysis

In this study, data were analyzed by META using STATA 17.0 software (Stata Corp LLC, College Station, TX, United States), with outcome indicators as continuous variables. This NMA integrated the before and after changes in the experimental and control groups in order to systematically assess the effects of different non-invasive methods on body composition, physical function and quality of life indicators in elderly patients with sarcopenia. To accurately assess the effects of these interventions, we calculated the standardized mean difference (SMD) and its 95% confidence intervals (CI) for each indicator, with a uniformly adjusted baseline of α = 0.05 and based on a random-effects model combined effect estimates to account for heterogeneity between studies in terms of participant characteristics and intervention modalities. Heterogeneity was quantified using the I^2^ statistic and Cochran’s Q test. Relationships between different non-invasive methods were visualized by means of network diagrams, where lines connecting nodes represent direct comparisons between different non-invasive methods. The size of the nodes and the thickness of the connecting lines were proportional to the number of studies that included that comparison, and this graphical presentation visualized the relative strengths of the interventions and their position in the network. In addition, the mapped network contributions further quantify the contribution of each direct comparison to the overall network, helping to analyze the influence of each intervention across the network. In addition, to assess publication bias in the study, publication bias for the main outcome indicators was analyzed using corrected comparison funnel plots. Finally, the probability of being the best intervention was calculated using the Surface Under the Cumulative Ranking Curve (SUCRA) method.

## 3 Result

### 3.1 Study selection

**FIGURE 1:**
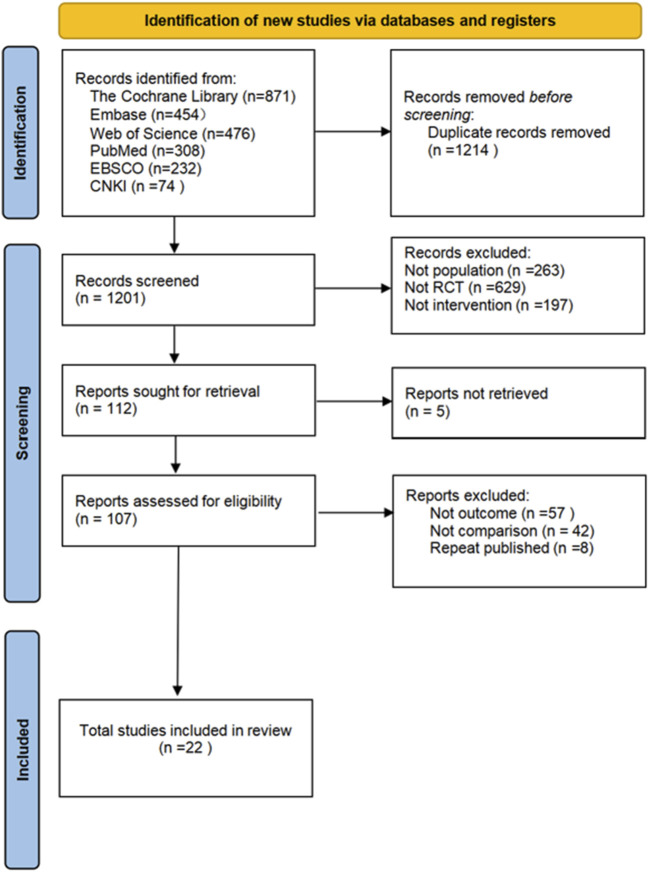
Literature search flowchart.

The flowchart for study selection is shown in Figure 1. A total of 2415 articles potentially eligible for the study were collected from different databases. To ensure the accuracy of the study and to avoid double counting of the same content, 1214 duplicate articles were removed through automatic and manual checking. The remaining 1201 articles needed to be screened. By analyzing the title and abstract of each article, 1094 ineligible articles were removed to ensure that only the most relevant literature to the research objectives was retained. The full text of 107 articles was obtained and read, and their study design, sample size, methodological quality and results were assessed in detail, leading to the identification of 22 randomized controlled trials. These trials met the quality criteria set by the study. Five different non-invasive treatments were evaluated. Each step of the screening process followed a strictly standardized procedure to ensure the reliability and scientific validity of the results.

### 3.2 Features of the included studies

Twenty-two studies were finally included, and the basic characteristics of all the included studies are detailed in Table 1. These studies were published between 2013 and 2024 and were conducted in China, Brazil, the United Kingdom, France, Germany, Japan, and South Korea. A total of 1062 sarcopenia patients were included in this study, 539 in the experimental group and 523 in the control group. Demographic data reported included country, age, and gender. Non-invasive treatments included aerobic training, resistance training, resistance aerobic training,WB-EMS and whole-body electrical the mean duration of treatment for the different rehabilitation interventions was 15.86 weeks, with 81.82% of the studies reporting interventions lasting longer than 8 weeks.

**TABLE 1:** Basic features of the included studies.

Study	Country	Duration	Group	Sample size	Age (mean±[SD])	Exercise category	Frequency	Outcome
Gadelha et al. (2016)	Brazil	8Weeks	AT	69	66.8 ± 5.4	kettlebell training	60 min/day,twice/ week	PBF
			CON	64	67.3 ± 5.0	usual activities		
Jung et al. (2022)	Korea	12Weeks	AT	14	75.4 ± 4.50	circuit training	45–75 min/day, three times/week	ASM, FM
			CON	14	74.6 ± 5.8	No		
Magtouf et al. (2023)	France	16Weeks	AT	25	76.3 ± 3.5	Total Mobility Plus Program	60 min/day, three times/week	BF, HG, MGS
			CON	25	75.9 ± 5.4	no		
Qi et al. (2023)	Japan	12Weeks	AT	9	67.6 ± 5.2	aerobic training	35 min/days, three times/week	SMI, BFM, GS, UGS
			CON	11	66.9 ± 5.4	no		
Vasconcelos et al. (2016)	Brazil	10Weeks	RT	14	72 ± 4.6	progressive resistance exercise	1 h/day,twice/week	Strength, GV, SF-36
			CON	14	72 ± 3.6	no		
Kim et al. (2016)	Japan	12Weeks	RAT	35	81.4 ± 4.3	Resistance Aerobic training	60 min/days, twice/week	ASM, GS, UWS
			CON	34	81.1 ± 5.1	no		
Huang et al. (2017)	China	12Weeks	RT	18	68.9 ± 4.9	elastic bands	three times/week	SMI, PBF
			CON	17	69.5 ± 5.1	no		
Liao et al. (2017)	China	12Weeks	RT	25	66.4 ± 4.5	elastic band exercise	60 min/days, three times/week	FFM, GS, HG
			CON	21	68.4 ± 5.9	no		
Park et al. (2017)	Japan	24Weeks	RAT	25	73.5 ± 7.1	resistance and aerobic training	50–80 min/day, 5 times/week	PBF, ASM, MWS, GS
			CON	25	74.7 ± 5.1	no		
Liao et al. (2018)	China	12Weeks	RT	33	66.7 ± 4.5	elastic band exercise	55 min/days, three times/week	PBF, TSM, GV
			CON	23	68.3 ± 6.1	no		
Chen et al. (2018)	China	8Weeks	RAT	17	66.7 ± 5.3	kettlebell training	60 min/day,twice/ week	SMM, BFM, HG
			CON	16	68.3 ± 2.8	no		
Fang et al. (2023)	China	12Weeks	RAT	29	73.8 ± 7.2	resistance and aerobic training	60 min/day, three times/week	TM, GS, BFM
			CON	30	73.8 ± 6.7	no		
Ferhi et al. (2023)	France	24Weeks	RT	20	76.6 ± 5.6	motricity exercises	60 min/day,twice/ week	BF, 2min-walk
			CON	20	74.1 ± 3.7	no		
Balachandran et al. (2014)	British	15Weeks	RAT	8	71.6 ± 7.8	combination training	twice/week	SPPB, GS, SMI, PBF
			RT	9	71.0 ± 8.2	Resistance training	twice/week	
Chen et al. (2017)	China	8Weeks	RAT	15	68.5 ± 2.7	combination training	each training mode: once/week	BFM, GS, SMM
			AT	15	68.9 ± 4.4	aerobic training	twice/week	
Wang et al. (2019)	China	8Weeks	AT	20	64.2 ± 3.0	aerobic training	30 min/day,twice/ week	ASM, PBF
			RAT	20	63.6 ± 5.2	resistance and aerobic training	60 min/day,twice/ week	
Zhou et al. (2018)	China	12Weeks	ES&P	23	70.4 ± 5.4	electric stimulator and aa	20min	WC
			CON	25	68.8 ± 5.1	no		
Kemmler and von Stengel (2013)	Germany	48Weeks	ES	23	74.7 ± 3.9	WB-EMS	18 min/day, three times/week	BF, SMM, GS, GV
			CON	23	74.7 ± 3.9	no		
Wittmann et al. (2016)	Germany	26Weeks	ES	25	76.4 ± 2.9	WB-EMS and protein supplementation	11–20 min/day, once/week,40 g/ day	BFM, ASM
			CON	25	77.4 ± 4.9	no		
Kemmler et al. (2016)	Germany	26Weeks	ES&P	25	76.4 ± 2.9	WB-EMS and protein supplementation	once/week	ASM, ABF
			ES	25	77.3 ± 4.9	WB-EMS	once/week	
Kemmler et al. (2016)	Germany	16Weeks	ES	34	78.1 ± 2.7	WB-EMS	14–20 min/day, three times/week	TBF, GS
			CON	34	76.9 ± 2.6	no		
Kemmler et al. (2018)	Germany	16Weeks	ES&P	33	77.1 ± 2.3	WB-EMS and protein supplementation	WB-EMS:14–20 min/ day, three times/week	TBF
			ES	33	78.1 ± 2.7	WB-EMS	P:1.7–1.8 g/kg/day	

CON, control group; AT, aerobic training; RT, resistance training; RAT, resistance-aerobic training; PBF, percent body fat; ASM, appendicular skeletal muscle mass; FM, fat mass; BF, body fat; HG, handgrip; MGS, maximum grip strength; SMI, skeletal muscle index; BFM, body fat mass; GS, grip strength; UGS, usual gait speed; GV, gait velocity; FFM, fat-free mass; TSM, total skeletal muscle mass; SMM, skeletal muscle mass; MWS, maximum walking speed; UWS, usual walking speed.

### 3.3 Risk of bias assessment

**FIGURE 2:**
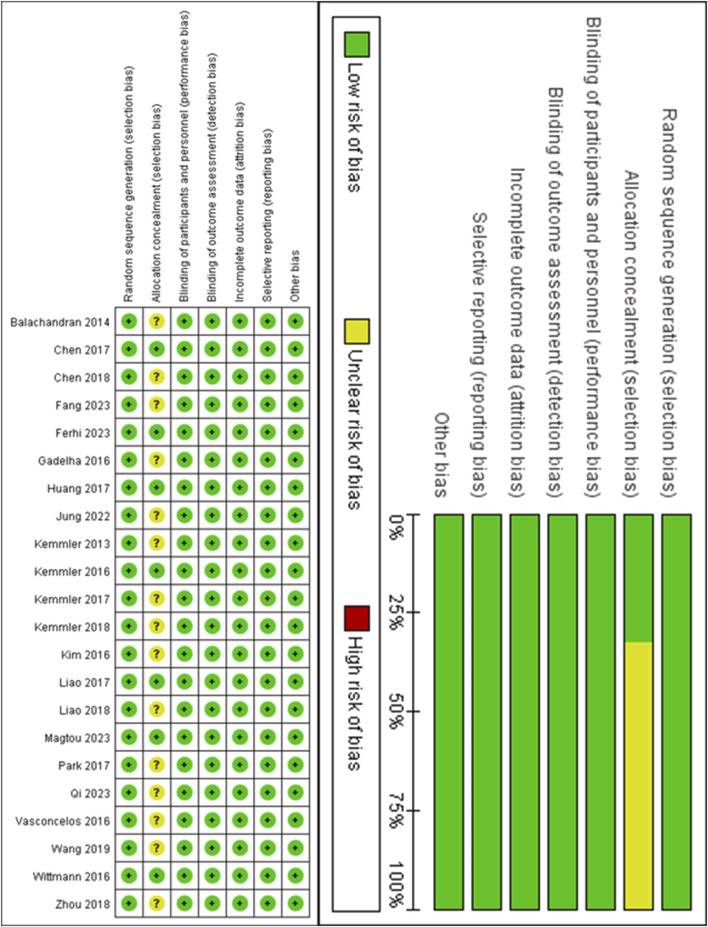
Evaluation results of literature quality risk bias of included studies.

Detailed information on the ROB assessment for each study is provided in Figure 2. A total of 22 articles mentioned random allocation, with 8 articles specifying the method of random allocation; 3 stated allocation concealment; 14 reported blinding; 14 reported blinding of outcome assessment; 14 studies demonstrated low risk of selective reporting; and all articles were free of other biases. In summary, 14 articles were judged to have a low ROB.

### 3.4 Direct pairwise meta-analyses

**FIGURE 3:**
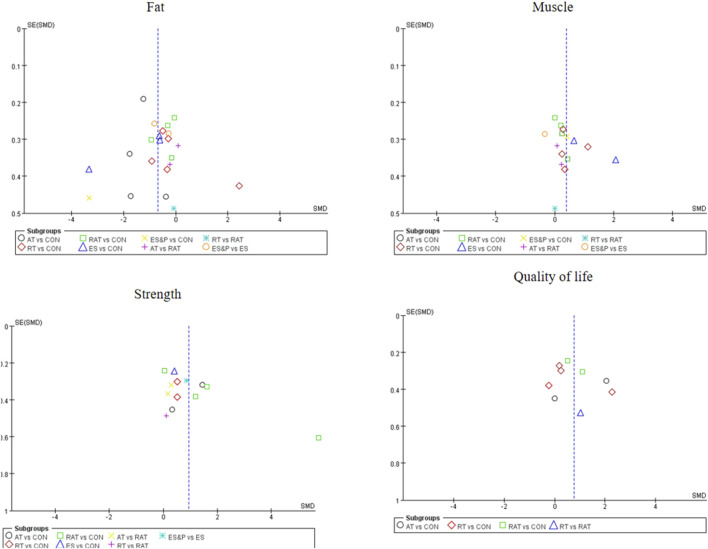
Funnel plot of fat, muscle, strength and quality of life in pairwise meta-analysis.

The funnel plot for pairwise comparisons is shown in Figure 3.

#### 3.4.1 Primary outcome

**FIGURE 4:**
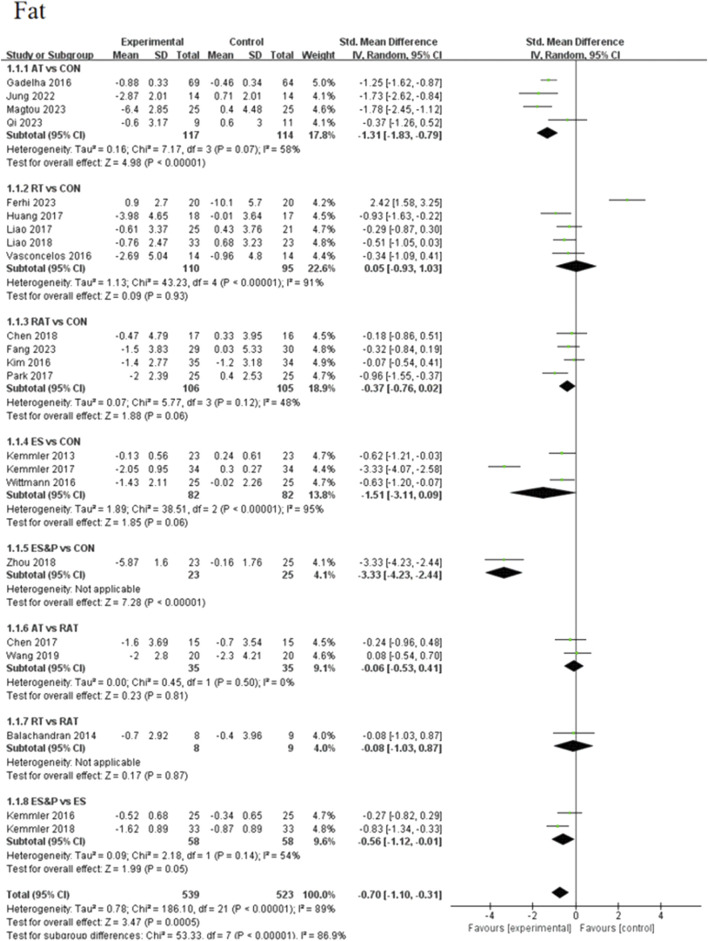
Forest plot of primary outcome (Fat).

**FIGURE 5:**
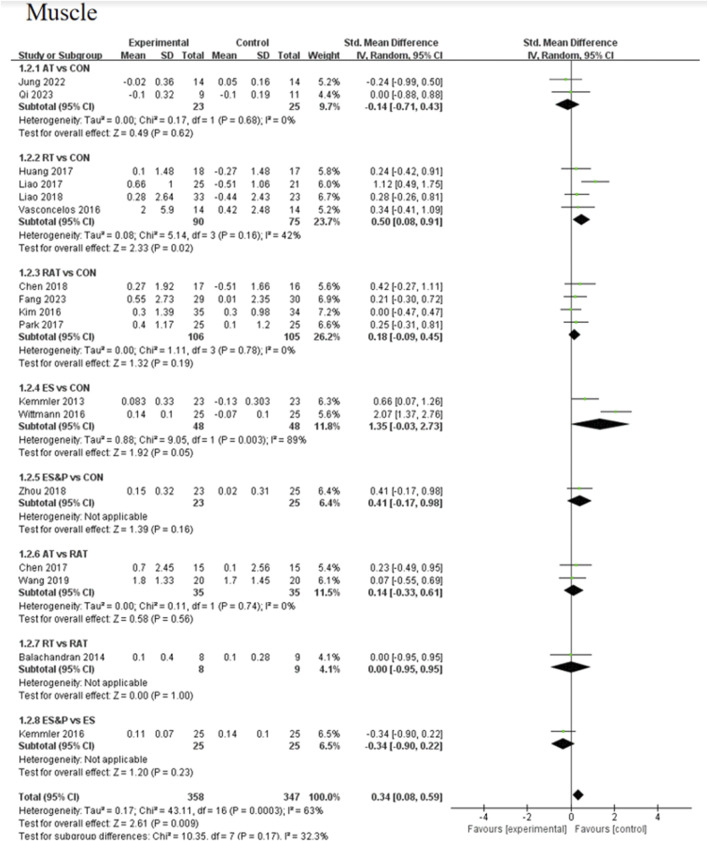
Forest plot of primary outcome (Muscle).

Forest plots of pairwise comparisons demonstrating the effects of different non-invasive therapies on fat and muscle are presented in Figures 4, 5. Compared to the control group, AT showed a significant effect in reducing fat, (SMD = −1.31, 95% CI [−1.83, −0.79], p < 0.00001, I^2^ = 58%), with a moderate degree of heterogeneity and small variability between findings; the ES&P group likewise showed a significant effect in reducing fat (SMD = −3.33, 95% CI [−4.23, −2.44], p < 0.00001); RT had a modest but non-significant effect in reducing fat (SMD = 0.05, 95% CI [−0.93, 1.03, p > 0.05, I^2^ = 91%) suggesting variability of results across studies; similarly, RAT did not demonstrate a significant advantage over the control group (SMD = −0.37, 95% CI [−0.76, 0.02], p > 0.05, I^2^ = 48%), suggesting that the effect of the therapy varied across studies, but the overall effect remained robust; there was also no significant difference in the effect of the ES group in lowering fat (SMD = −1.51, 95% CI [−3.11, 0.09], p > 0.05 I^2^ = 95%), suggesting differences in results across studies. The difference in effect on reducing fat between the AT and RAT groups was not significant, (SMD = −0.06, 95% CI [−0.53, 0.41], p > 0.05, I^2^ = 0%) suggesting a high degree of consistency of effect across studies; the effect of RT compared to the RAT group on reducing fat was also not significant (SMD = −0.08, 95% CI [−1.03, 0.87], p > 0.05).The ES&P group had a better effect in the former compared to the ES group, but the difference was not significant (SMD = −0.56, 95% CI [−1.12, −0.01], p = 0.05, I^2^ = 54%), suggesting a moderate degree of variability between their studies.

The AT group showed a non-significant effect in increasing muscle compared to the control group (SMD = −0.14, 95% CI [−0.71, 0.43], p > 0.05, I^2^ = 0%), suggesting a high degree of consistency of effect across studies; the RT group achieved a significant effect in increasing muscle compared to the control group (SMD = 0.50, 95% CI [0.08, 0.91], p < 0.05, I^2^ = 42%), suggesting that the effect of the therapy fluctuated across studies but was generally robust; similarly, the RAT group did not show a significant advantage over the control group (SMD = 0.18, 95% CI [−0.09, 0.45], p > 0.05, I^2^ = 0%), with a high degree of consistency of results between studies; in the case of a comparison of the AT group and the RAT group and in the comparison between RT and RAT groups, no significant differences were shown either. The ES group had an effect in terms of muscle gain, but this was also not significant (SMD = 1.35, 95% CI [−0.03, 2.73], p = 0.05, I^2^ = 89%), suggesting differences between studies; nor was there a significant effect in the ES&P group in relation to the control group advantage (SMD = 0.41, 95% CI [−0.17, 0.98], p > 0.05); similarly the ES&P group did not show a significant difference from the ES group (SMD = −0.34, 95% CI [-0.90, 0.22], p > 0.05).

#### 3.4.2 Secondary outcomes

**FIGURE 6:**
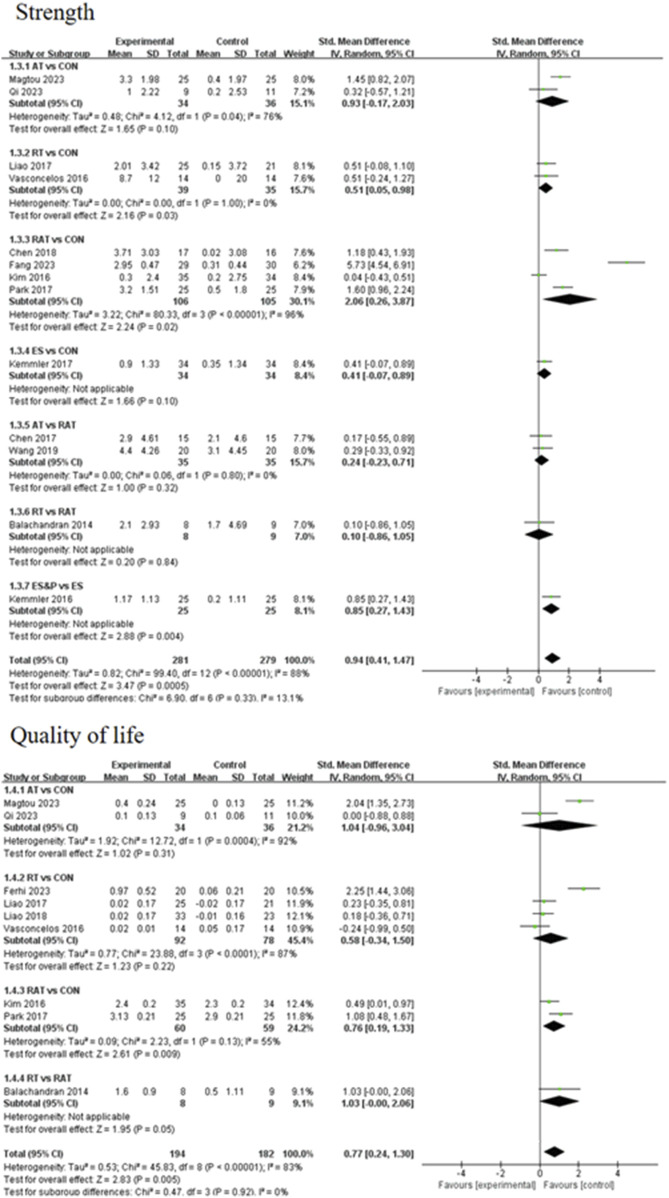
Forest plot of secondary outcomes.

A forest plot of secondary outcomes based on non-invasive treatments is shown in Figure 6. The results of the two-by-two meta-analysis showed that RT was significantly effective in improving muscle strength (SMD = 0.51, 95% CI [0.05, 0.98], p < 0.05, I^2^ = 0%), with a high degree of agreement across studies. In addition, RAT also showed a significant effect in improving muscle strength (SMD = 2.06, 95% CI [0.26, 3.87], p < 0.05, I^2^ = 96%), but the results varied between studies. The difference between RT and RAT groups was not significant (SMD = 0.10, 95% CI [-0.86, 1.05], p > 0.05). Other interventions (AT and ES) were not significant compared to the control group. ES&P group showed better efficacy compared to ES group and the difference was significant (SMD = 0.85, 95% CI [0.27, 1.43], p < 0.001).

For improving patients’ quality of life, the efficacy of RAT was more significant compared to the control group (SMD = 1.42, 95% CI [0.13, 2.70], p < 0.05, I^2^ = 55%), with moderate inter-study variability. Other intervention efficacies were not significant.

### 3.5 Network meta-analysis

#### 3.5.1 Network diagram of included studies

**FIGURE 7:**
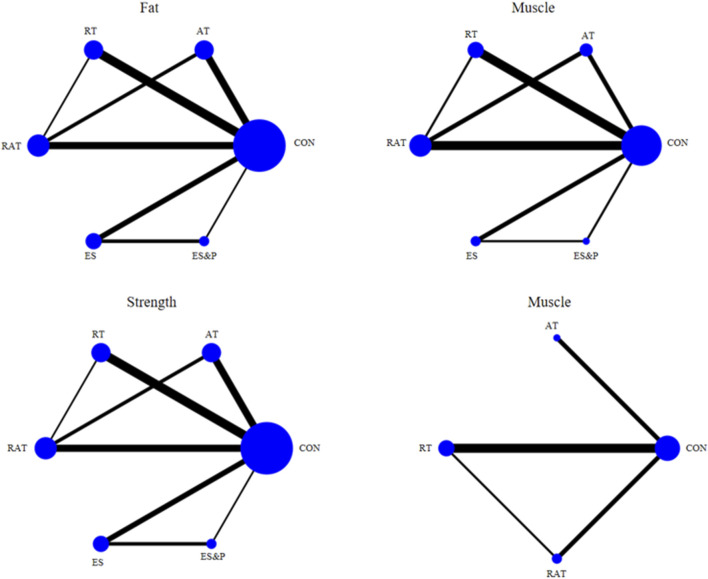
Network plot of outcome indicators.

The six dots in the figure represent the six interventions, the straight lines between the dots represent the existence of a direct comparison between the interventions, and the thickness of the line represents the number of direct comparisons between the two interventions. Except for the quality-of-life indicator, which was 4 interventions, all the outcome indicators were 6 interventions (including the control group) and included the same interventions. Interventions in the experimental group included AT, RT, RAT, ES, and ES&P; the control group was a non-combined group, with RAT being the most widely researched intervention and fewer studies on ES&P. The Network diagram of the outcome indicators is detailed in Figure 7.

#### 3.5.2 Ranking of intervention effectiveness of five non-invasive treatments

**FIGURE 8:**
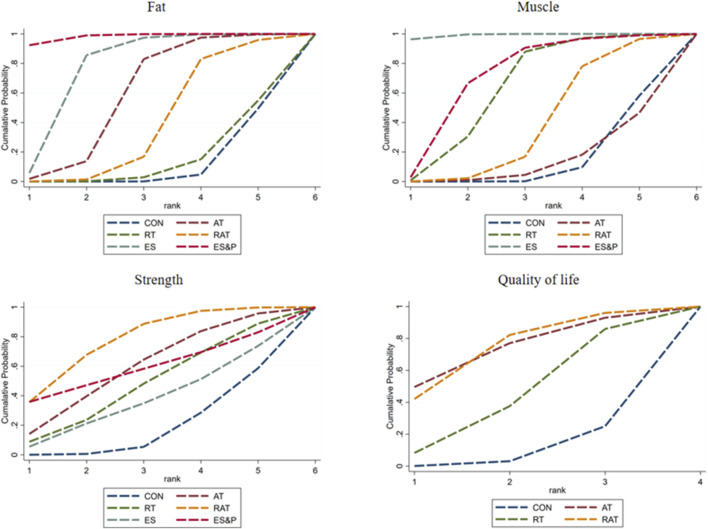
Ranking of intervention effects for outcome indicators.

FAT METRICS: The effectiveness of the five non-invasive treatments for fat reduction in senile sarcopenia was ranked as ES&P ([SUCRA] = 98.2), ES ([SUCRA] = 77.7), AT ([SUCRA] = 59.1), RAT ([SUCRA] = 39.4), and RT ([SUCRA] = 14.6) were better than that of the no intervention control CON ([SUCRA] = 10.9), as detailed in Figure 8; Tables 2, 3.

**TABLE 2:** Ranking of the probability of improving fat, muscle, strength and quality of life class in elderly sarcopenia by five non-invasive treatments.

Treatment	Fat		Muscle		Strength		Quality of life	
	SUCRA (%)	Rank	SUCRA (%)	Rank	SUCRA (%)	Rank	SUCRA (%)	Rank
CON	10.9	6	13.6	6	18.6	6	8.7	4
AT	59.1	3	14.0	5	59.6	2	73.8	1
RT	14.6	5	63.2	3	47.8	4	44.1	3
RAT	39.4	4	38.7	4	77.9	1	73.4	2
ES	77.7	2	99.2	1	37.3	5		
ES&P	98.2	1	71.2	2	58.8	3		

**TABLE 3:** Network meta-analysis matrix of outcome.

Fat					
ES&P	0.87 (−0.23,1.98)	2.03 (0.57,3.50)	2.55 (1.07,4.02)	1.57 (0.08,3.06)	2.58 (1.32,3.84)
−0.87 (−1.98,0.23)	ES	1.16 (−0.05,2.38)	1.67 (0.44,2.91)	0.69 (−0.56,1.95)	1.71 (0.75,2.68)
−2.03 (−3.50, −0.57)	−1.16 (−2.38,0.05)	RAT	0.51 (−0.48,1.50)	−0.47 (−1.37,0.44)	0.55 (−0.19,1.29)
−2.55 (−4.02, −1.07)	−1.67 (−2.91, −0.44)	−0.51 (−1.50,0.48)	RT	−0.98 (−2.06,0.11)	0.04 (−0.73,0.81)
−1.57 (−3.06, −0.08)	−0.69 (−1.95,0.56)	0.47 (−0.44,1.37)	0.98 (−0.11,2.06)	AT	1.02 (0.22,1.82)
−2.58 (−3.84, −1.32)	−1.71 (−2.68, −0.75)	−0.55 (−1.29,0.19)	−0.04 (−0.81,0.73)	−1.02 (−1.82, −0.22)	CON

Muscle					
ES&P	0.55 (−0.01,1.10)	−0.42 (−1.06,0.21)	−0.16 (−0.82,0.51)	−0.65 (−1.37,0.07)	−0.62 (−1.18, −0.06)
−0.55 (−1.10,0.01)	ES	−0.97 (−1.54, −0.40)	−0.70 (−1.31, −0.10)	−1.19 (−1.86, −0.53)	−1.16 (−1.65, −0.68)
0.42 (−0.21,1.06)	0.97 (0.40,1.54)	RAT	0.27 (−0.19,0.72)	−0.22 (−0.67,0.22)	−0.19 (−0.50,0.12)
0.16 (−0.51,0.82)	0.70 (0.10,1.31)	−0.27 (−0.72,0.19)	RT	−0.49 (−1.06,0.08)	−0.46 (−0.82, −0.10)
0.65 (−0.07,1.37)	1.19 (0.53,1.86)	0.22 (−0.22,0.67)	0.49 (−0.08,1.06)	AT	0.03 (−0.43,0.49)
0.62 (0.06,1.18)	1.16 (0.68,1.65)	0.19 (−0.12,0.50)	0.46 (0.10,0.82)	−0.03 (−0.49,0.43)	CON

Strength					
ES&P	−0.85 (−3.81,2.10)	0.44 (−3.91,4.79)	−0.44 (−4.97,4.09)	−0.07 (−4.54,4.39)	−1.26 (−5.42,2.91)
0.85 (−2.10,3.81)	ES	1.29 (−1.90,4.49)	0.41 (−3.02,3.84)	0.78 (−2.58,4.13)	−0.41 (−3.34,2.53)
−0.44 (−4.79,3.91)	−1.29 (−4.49,1.90)	RAT	−0.88 (−2.81,1.05)	−0.52 (−2.14,1.10)	−1.70 (−2.96, −0.44)
0.44 (−4.09,4.97)	−0.41 (−3.84,3.02)	0.88 (−1.05,2.81)	RT	0.37 (−1.93,2.66)	−0.82 (−2.60,0.96)
0.07 (−4.39,4.54)	−0.78 (−4.13,2.58)	0.52 (−1.10,2.14)	−0.37 (−2.66,1.93)	AT	−1.18 (−2.80,0.44)
1.26 (−2.91,5.42)	0.41 (−2.53,3.34)	1.70 (0.44,2.96)	0.82 (−0.96,2.60)	1.18 (−0.44,2.80)	CON

Quality of life					
RAT	0.99 (−0.18,2.16)	0.47 (−0.44,1.38)	1.06 (−0.35,2.47)		
−0.99 (−2.16,0.18)	RT	−0.52 (−1.82,0.79)	0.07 (−1.76,1.90)		
−0.47 (−1.38,0.44)	0.52 (−0.79,1.82)	AT	0.59 (−1.09,2.26)		
−1.06 (−2.47,0.35)	−0.07 (−1.90,1.76)	−0.59 (−2.26,1.09)	CON		

Muscle indicator: The effectiveness of the five non-invasive treatments for muscle mass increase in senile sarcopenia was ranked as ES ([SUCRA] = 99.2), ES&P ([SUCRA] = 71.2), RT ([SUCRA] = 63.2), RAT ([SUCRA] = 38.7), and AT ([SUCRA] = 14.0) being superior to the control group CON ([SUCRA] = 10.9), which had no intervention, and the control group CON ([SUCRA] = 10.9), which had no intervention, as detailed in Figure 8; Tables 2, 3. CON ([SUCRA] = 13.6) in the control group without any intervention, as shown in Figure 8; Tables 2, 3.

Strength indicators: The effectiveness of the five non-invasive treatments in improving strength in old age sarcopenia was ranked as RAT ([SUCRA] = 77.9), AT ([SUCRA] = 59.6), ES&P ([SUCRA] = 58.8), RT ([SUCRA] = 47.8), and ES ([SUCRA] = 37.3) were better than the control group CON ([SUCRA] = 13.0) without any intervention, as detailed in Figure 8; Tables 2, 3. ([SUCRA] = 18.6), which were better than CON ([SUCRA] = 18.6), the control group without any intervention, as shown in Figure 6; Tables 2, 3.

Quality of life: The effectiveness of the three non-invasive treatments in increasing QoL in old age sarcopenia was ranked as AT ([SUCRA] = 73.8), RAT ([SUCRA] = 73.4), and RT ([SUCRA] = 44.1) were better than the control group CON ([SUCRA] = 8.7) who did not have any interventions, as shown in Figure 8; Table 2, and Table 3.2. Table 3.

### 3.6 Small sample effect or publication bias test

**FIGURE 9:**
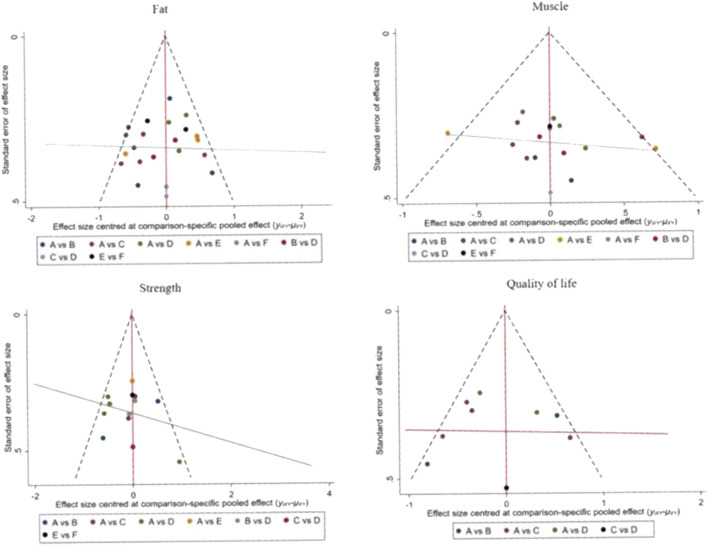
Corrected comparison funnel plot for outcome indicators. A = CON; B = AT; C = RT; D = RAT; E = ES; D = ES&P.

For studies included in the reticulated META analyses, small-sample effect estimates and publication bias tests were performed using corrected-comparison funnel plots. The included studies were largely symmetrical, suggesting that there was no small-sample effect in the current study, and no significant publication bias was found. See Figure 9.

## 4 Discussion

### 4.1 Analysis of the efficacy of five non-invasive treatments on the main outcome indicators of body composition, physical function and quality of life in elderly patients with sarcopenia

In this study, we analyzed the effects of five non-invasive treatments on body composition, physical functioning and quality of life in elderly patients with sarcopenia, focusing in particular on changes in body composition-related indicators fat (fat) and muscle (muscle). The results of the study showed that, targeting fat reduction, the ES& P group demonstrated the most significant effect in reducing fat (SMD = −3.33, 95% CI [−4.23, −2.44], p < 0.00001), which was significantly better than the other treatment groups. These results support the significant role of ES& P intervention in reducing body fat in elderly patients with sarcopenia. However, the RT group did not demonstrate a significant effect in fat reduction, suggesting differences in efficacy of fat reduction between treatments. In contrast, clinical research demonstrates that NMES combined with high-protein supplementation can effectively slow the progression of muscle atrophy in patients (Verceles et al., 2023). Resistance training demonstrates significant dual efficacy in older adults, concurrently promoting muscle hypertrophy and facilitating fat mass reduction (Vikberg et al., 2019). The high heterogeneity between the RT and ES groups in this study may reflect individual differences as well as diversity in therapy implementation. Therefore, multimodal therapies combining exercise and nutritional interventions may be more desirable treatment options in clinical practice, especially in targeting the effects on fat loss.

In terms of the effect on increasing muscle, RT (SMD = 0.50, p < 0.05) demonstrated a significant improvement, with an increase compared to the control group. This is consistent with Kemmler’s (Kemmler et al., 2020) study that resistance exercise maintains lumbar muscle strength and improves leg extensor strength. However, the other treatments, especially the AT, RAT and ES groups did not reach significant levels. In particular, neither the ES group (SMD = 1.35, p = 0.05) nor the ES&P group (SMD = 0.41, p > 0.05) showed a significant advantage over the control group. This is in line with existing research. Roschel (Roschel et al., 2021) showed that training combined with different supplements did not increase the original training effect. The AT and RAT groups showed more consistent results, although not statistically significant improvements in muscle mass. However, Ikeda demonstrated that combined interventions may represent a feasible and effective approach for increasing skeletal muscle mass (Ikeda et al., 2023).

### 4.2 Analysis of the efficacy of five non-invasive treatments on body composition, physical function, and secondary outcome indicators of quality of life in elderly patients with sarcopenia

In this study, addressing the effects of five non-invasive therapeutic interventions on body composition, function and quality of life in elderly patients with sarcopenia, we found significant differences between treatments in improving muscle strength and quality of life. A NMA of randomized controlled trials showed that RAT treatment was the most effective in improving muscle strength, followed by AT, ES&P, RT and ES, all of which were superior to the control group (CON). These results reflect the effectiveness of different non-invasive treatments in improving strength in elderly patients with sarcopenia, with RAT having the most significant effect ([SUCRA] = 77.9) and showing high consistency of effect across multiple studies (SMD = 2.06, 95% CI [0.26, 3.87], p < 0.05).

Further analyses showed that RT treatment also demonstrated a significant effect in improving muscle strength (SMD = 0.51, 95% CI [0.05, 0.98], p < 0.05) and a high degree of consistency of effect across studies, suggesting a high degree of reproducibility and reliability of RT. This is consistent with Hurst’s findings (Hurst et al., 2022) that resistance training is effective in improving muscle strength and muscle mass. However, there was no significant difference between the effects of RAT and RT in improving muscle strength (SMD = 0.10, 95% CI [−0.86, 1.05], p > 0.05), and there may be some basis for selection between the two for clinical application.AT and ES treatments did not show a significant effect in this study compared with the control group, suggesting that the improvement of muscle strength with these treatments may be more limited or more individualized intervention programmers may be required. This is consistent with the findings of Dehail et al., 2008) that ES is not superior to traditional treatments.

Regarding quality of life assessment measured by physical performance indicators (grip strength, GS, MWS, and 2-min walk test), the NMA demonstrated AT and RAT achieved the highest rankings (SUCRA = 73.8 and 73.4 respectively), with RT showing moderate efficacy (SUCRA = 44.1). These functional improvements, while not assessing psychological or social QoL domains, align with Dianatinasab (Dianatinasab et al., 2020) who reported similar physical capacity enhancements through exercise interventions. However, the ES treatment group performed the worst of all metrics, suggesting a possible weak efficacy in intervening on quality of life in patients with sarcopenia.

#### 4.2.1 Limitations

Several limitations should be acknowledged in this network meta-analysis. First, the high variability in treatment effects among multiple outcome indicators likely reflects differential biological mechanisms associated with diverse intervention approaches. Second, all studies had relatively small sample sizes, which may limit the statistical power to detect treatment differences. Third, quality of life was assessed indirectly through physical performance metrics rather than validated QoL questionnaires, potentially overlooking psychosocial dimensions. Future studies should employ larger sample sizes, incorporate validated QoL assessments, and explore biological mechanisms to address these limitations.

## 5 Conclusion

ES&P and AT had good results for fat, RT was the most effective for increasing muscle, RAT was the most effective for improving strength, and AT was the best for improving quality of life. These findings suggest that different non-invasive treatments have some differences in their efficacy on different functional indicators. Therefore, in clinical practice, multiple treatments should be individually selected and combined according to the patient’s specific type of dysfunction as well as their overall physical condition in order to achieve optimal functional recovery. Such personalized treatment plans not only target the specific problems of the patient, but also improve the effectiveness of the treatment and the quality of life of the patient. Future research should delve deeper into the long-term effects of these non-invasive intervention methods and identify the most appropriate treatment options and parameter configurations through systematic analyses, thus providing patients with more precise and efficient treatment options. In addition, as technology and research continue to progress, exploring new treatment methods and the integrated application of different intervention approaches will also be a key direction for future research.

## Abbreviations

GV, Gait velocity; PBF, Percentage body fat; ASM, appendicular skeletal muscle mass; MWS, maximum walking speed; UWS, Usual walking speed; GS, Gait velocity; TSM, total skeletal muscle mass; FFM, fat-free mass; HG, Hand grip; SMM, skeletal muscle mass; BFM, body fat mass; SMI, SMI (%)Skeletal Muscle mass Index; UGS, Usual gait speed; BF, BF (%) body fat; TBF, Total body fat mass; FM, fat mass; WC, Waist circumference; ABF, Abdominal body fat; TM, Total muscle mass; AT, aerobic training; RT, resistance training; RAT, resistance and aerobic training; ES, electrical stimulation; ES&P, electrical stimulation and protein supplementation; WB-EMS, whole-body electrical stimulation; NMES, neuromuscular electrical stimulation.
